# Liquid Bread and Invisible Worlds—Beer, Fungi, and the Origins of Microbial Control

**DOI:** 10.3201/eid3209.AC3209

**Published:** 2026-09

**Authors:** Nicolas Hoffmann

**Affiliations:** Marist School, Atlanta, Georgia, USA; Georgia Gwinnett College, Lawrenceville, Georgia, USA

**Keywords:** fungi, art-science connection

**Figure:**
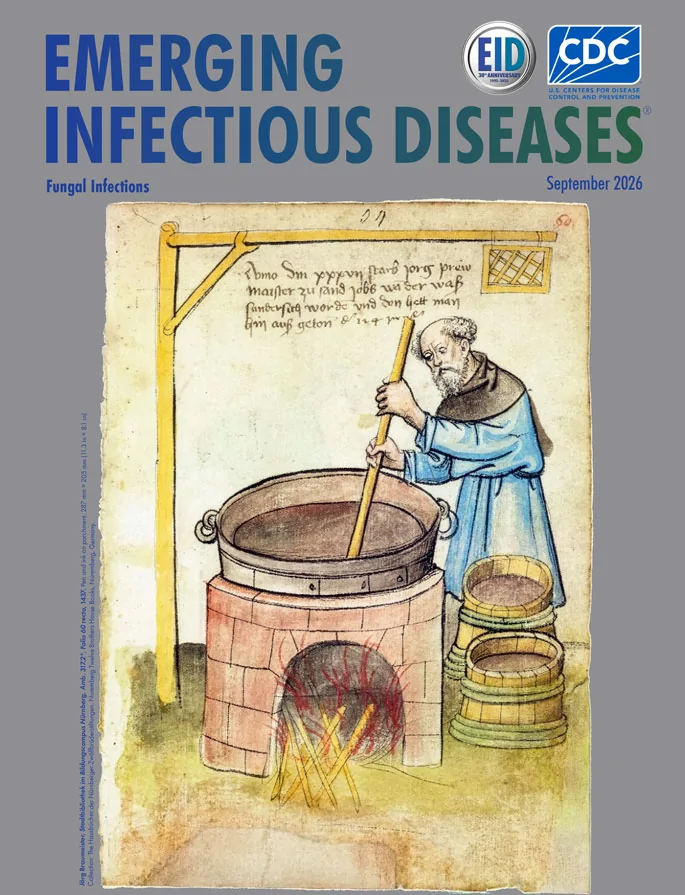
**Jörg Braumeister, *Stadtbibliothek im Bildungscampus Nürnberg, Amb. 317.2°, Folio 60 recto*,** 1437. Pen and ink on parchment. 287 mm × 205 mm (11.3 in × 8.1 in). Collection: The Hausbücher der Nürnberger Zwölfbrüderstiftungen. Nuremberg Twelve Brothers House Books, Nuremberg, Germany.

Beer is among humanity’s oldest inventions. Academic debate continues over what came first, beer or bread, and one leading hypothesis suggests beer itself evolved from earlier accidents: perhaps from grain stores that became wet and spontaneously fermented, or from observations of mead, when honey and rainwater were colonized by wild yeasts. Whatever the precise origin, the result was the same. Humans began cultivating a process they did not yet understand.

This month’s cover image captures that prescientific moment. A lone figure stands before fire and liquid, engaged in a process both ordinary and profound—the production of a beverage essential for hydration, nutrition, and safety in medieval life. The brewer does not know it, but the labor is shared with unseen fungal partners.

The illustration comes from the *Hausbücher der Nürnberger Zwölfbrüderstiftungen* (Nuremberg Twelve Brothers House Books, Nuremberg, Germany), a set of illuminated manuscripts compiled during 1426–1806 to document the trades of craftsmen supported by the Mendel and Landauer charitable foundations in Nuremberg. Each folio depicts a single artisan at work, accompanied by a brief biographical entry. The illustrators are largely anonymous, and individual attribution is rarely possible. The manuscripts are held by the Stadtbibliothek Nürnberg and have been fully digitized (https://www.nuernberger-hausbuecher.de).

The brewer here is a layman—not, despite popular association, a monk—supported by the foundation in old age. According to the description on the obverse, “Jörg Breu was the first almshouse brother of whom it is recorded—according to the inscription—that he was discharged from the Twelve Brothers’ House due to illness; in this instance, he was transferred to the St. Jobst Leper Hospital.”

He stirs a heated vessel while smaller tubs await the finished product, the fire beneath carefully controlled. The scene conveys method, repetition, and accumulated knowledge. Yet it also reflects a world in which craft knowledge far outpaced scientific explanation. Brewers understood the process; they did not understand the how.

For most of human history, fermentation was observed rather than understood. Grain was soaked, bread dissolved, liquid boiled, and time did the rest. What transformed that mixture was not magic but fungi. Yeast, single-celled fungi of the genus *Saccharomyces*, consume sugars and produce alcohol and carbon dioxide, converting grain into something stable, caloric, and safer to drink. But yeast do not act alone. They exist within a microbial ecosystem that includes bacteria and other fungi; some contribute desirable flavors, others spoil the batch, and still others, introduced through contaminated water or poorly managed fermentation, can cause illness.

The fungal kingdom occupies a peculiar place in human history, both intimate and dangerous. Fungi make possible some of humanity’s most important foods—bread, beer, cheese—yet they are also notable agents of disease. Recent estimates attribute ≈3.8 million deaths annually to invasive fungal infection, and roughly 2.5 million are directly caused by fungal pathogens such as *Candida*, *Aspergillus*, *Cryptococcus*, and *Pneumocystis*. In 2022, the World Health Organization issued its first fungal priority pathogens list, recognizing rising antifungal resistance and the scarcity of diagnostic and therapeutic tools. Mycotoxins compound the burden; ergot alkaloids in contaminated grain, for example, have historically caused outbreaks of mass poisoning and hallucination.

To premodern populations, such outcomes were indistinguishable in cause. The same unseen forces that produced nourishment could also produce sickness. Beer sat at that intersection, a product of controlled fungal activity whose safety depended on keeping other microbial actors in check.

By the early 16th Century, brewers in the German states attempted to impose order on this unpredictable system. The Reinheitsgebot of 1516 limited beer ingredients to water, grain, and hops. Notably absent was yeast, which, although essential, remained invisible to science. The law is often remembered as a cultural constraint, but in practice it functioned as risk reduction. Brewers had long experimented with additives—some harmless, others toxic—and by restricting ingredients, the law lowered the likelihood of contamination, poisoning, and unintended fermentation. Hops in particular contain antimicrobial compounds that inhibit bacterial growth, helping stabilize beer and extend its shelf life. Without knowing it, brewers were practicing infection control.

Beer’s role in survival reaches even further back. In ancient Egypt, laborers were partly compensated in beer rations: thick, nutritious, and calorically dense, fermented from bread and grain. That early beer was, quite literally, liquid bread, and it offered a crucial advantage: relative safety. In environments where waterborne pathogens were common, fermentation, boiling, alcohol, and acidity all inhibited microbial growth. Beer was not sterile, but it was often safer than the available water.

That relationship changed in the 19th Century, when Louis Pasteur demonstrated that fermentation was driven by living microorganisms, specifically, by yeast. Yeast could now be isolated, cultivated, and standardized; contamination could be identified and spoilage prevented with intention. What had once been an uncertain collaboration became a controlled partnership.

The brewer in this image stands at a moment before germ theory, before microbiology, before fungi were understood as either allies or pathogens. Yet he works within that system reliably, and with consequences that continue to shape human health. Beer represents one of humanity’s earliest and most successful negotiations with fungi, harnessing some organisms to create stability while suppressing others that might cause harm. It is a story not of elimination but of coexistence, and one that remains unfinished. As antifungal resistance grows and invasive mycoses claim millions of lives each year, the brewer’s vat reminds us that managing fungi has always been, and remains, a public health enterprise.
